# Failure of self-consistency in the discrete resource model of visual working memory

**DOI:** 10.1016/j.cogpsych.2018.05.002

**Published:** 2018-09

**Authors:** Paul M. Bays

**Affiliations:** University of Cambridge, Department of Psychology, Cambridge CB2 3EB, UK

**Keywords:** Short-term memory, Slot model, Resource model, Hybrid model, Precision

## Abstract

The discrete resource model of working memory proposes that each individual has a fixed upper limit on the number of items they can store at one time, due to division of memory into a few independent “slots”. According to this model, responses on short-term memory tasks consist of a mixture of noisy recall (when the tested item is in memory) and random guessing (when the item is not in memory). This provides two opportunities to estimate capacity for each observer: first, based on their frequency of random guesses, and second, based on the set size at which the variability of stored items reaches a plateau. The discrete resource model makes the simple prediction that these two estimates will coincide. Data from eight published visual working memory experiments provide strong evidence against such a correspondence. These results present a challenge for discrete models of working memory that impose a fixed capacity limit.

## Highlights

• Discrete resource models impose an upper bound on number of items stored.
• This capacity can be estimated either from recall variability or guessing frequency.
• We show that these two estimates of capacity do not coincide in published data.
• This finding challenges the validity of discrete models of working memory.

## 1 Introduction

Working memory, the ability to maintain information from the external world in an active internal state, is highly limited. Correctly characterizing this limitation is essential for understanding changes over the lifespan, exploring individual differences and for clinical assessment. Most early models assumed the limit could be adequately described by a fixed maximum number of objects retained at one time (Cowan, 2001, Luck and Vogel, 1997, Miller, 1956). However, it is now well established that the precision (resolution) with which information is stored declines monotonically with the number of items in memory (Bays and Husain, 2008, Palmer, 1990, Wilken and Ma, 2004). This finding is most straightforwardly accounted for by continuous resource models, which propose that a fixed quantity of a representational medium is shared out between items: precision of an item’s recollection is determined by the amount of resource allocated to it (Bays et al., 2009, Gorgoraptis et al., 2011, Ma et al., 2014, van den Berg et al., 2012). According to continuous resource models there is no fixed upper limit: instead, as the number of objects in memory increases, representational fidelity degrades until recall is indistinguishable from noise. A strong advantage of continuous resource models is their biological plausibility, and they have found support in neurophysiological data (Emrich et al., 2013, Sprague et al., 2014) and neurally-inspired models (Bays, 2014, Schneegans and Bays, 2017).

**Fig. 1:**
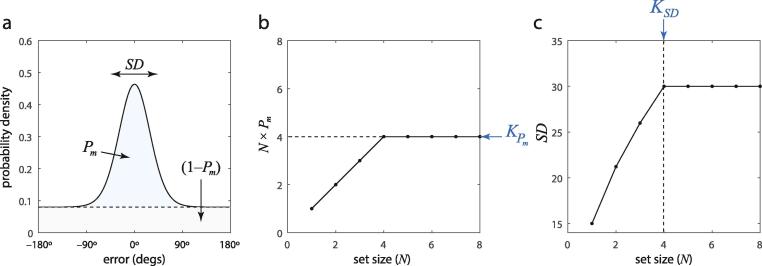
Two methods of estimating capacity, according to the discrete resource model. (a) Response errors arise from one of two distributions. When the item is in memory, with probability $P_{m}$, a response is drawn from a von Mises distribution (blue) with width *SD*. When the item is out of memory, with probability $1-P_{m}$, a response is drawn from a uniform (guessing) distribution (gray). (b) The number of items in memory, estimated by $N\times P_{m}$, reaches a maximum at the capacity limit, providing a capacity estimate $K_{P_{m}}$. (c) The width of the von Mises distribution, *SD* reaches a maximum and plateaus when the capacity limit is reached, providing a second capacity estimate $K_{\mathrm{SD}}$. (For interpretation of the references to color in this figure legend, the reader is referred to the web version of this article.)

An alternative viewpoint retains the concept of a fixed maximum number of items, but combines it with a resource or resource-like behavior below this capacity. Most prominently, Zhang and Luck (2008) proposed a “discrete resource” model, in which a fixed number of memory slots can be flexibly allocated to items, such that a single object can be stored multiple times, enhancing the precision of its recall. This conclusion was based on fitting a model in which responses were drawn from a mixture of two distributions: a normal (von Mises) distribution corresponding to noisy recall of an item in memory, and a uniform distribution corresponding to random guessing when the item tested is out of memory (Fig. 1a). The discrete resource model predicts that the capacity limit will affect the parameters of this fit in two ways: first, the mixture proportion of the normal component ($P_{m}$) should reflect the probability that an item is in memory, so the product of the set size with $P_{m}$ reaches a maximum at capacity (Fig. 1b); second, the standard deviation (*SD*) of the normal component should increase with set size until capacity is reached and then plateau (Fig. 1c).

This allows for a simple test of the self-consistency of the discrete resource model: capacity estimates calculated from $P_{m}$ and *SD* should be equal^1^. Here we tested this prediction and found it to be false, providing evidence against the concept of discrete representations in working memory.

## 2 Methods

### 2.1 Studies

We analysed data from eight studies that used the continuous reproduction method to test visual working memory recall (Bays, 2014, Bays et al., 2009, Gorgoraptis et al., 2011, Pratte et al., 2017, van den Berg et al., 2012, Wilken and Ma, 2004, Zhang and Luck, 2008). Four studies tested memory for color and four tested memory for orientation. Data from six of the eight studies were previously made public as part of the Ma lab benchmark data set (http://www.cns.nyu.edu/malab/resources.html); we included all (unretracted) studies from that data set in which at least four different set sizes were tested, including set size one. One additional study (Bays, 2014) was from the author’s own laboratory, and the final study (Pratte et al., 2017) was data originally made available to the author as part of another project.

### 2.2 Analysis

Following Zhang and Luck (2008) we obtained fits to response data, from each participant at each set size, of a model that assumed responses were generated from a mixture of two distributions, one von Mises (a circular analogue of the Gaussian) and one uniform:

(1) p(θ^)=PmϕSD(θ^-θ)+(1-Pm)12π,

where θ is the target feature value, θ^ is the reported feature value, and $P_{m}$ is the probability that the target item is in memory. $\phi_{\mathrm{SD}}(\cdot )$ denotes the probability density function of a von Mises with mean of zero and circular standard deviation *SD*. Maximum likelihood fits were obtained using an Expectation Maximization algorithm and a range of initial parameter values (code available at http://www.bayslab.com/code/JV10/).

To estimate capacity based on the frequency of guessing, for each subject we calculated an estimate of the number of items in memory at each set size, equal to the product of the set size, *N*, with $P_{m}(N)$, the estimated probability of remembering an item at that set size. We then took the maximum of these values as our estimate of capacity:

(2) $$K_{P_{m}}=\underset{N}{\max}\{N\times P_{m}(N)\}.$$

To estimate capacity based on the plateau in recall variability we again followed Zhang & Luck and fit a function relating *SD* at each set size to capacity:

(3) $$\mathrm{SD}(N)=\sigma_{1}\sqrt{\min\{N,K_{\mathrm{SD}}\}},$$

where $\sigma_{1}$, the variability at set size one, and $K_{\mathrm{SD}}$, the capacity, are free parameters. The logic behind this formula is that, when there are fewer items than capacity, multiple independent samples of each item can be obtained and averaged, with the result that the standard deviation of the average is inversely proportional to the square root of the number of samples (which is inversely proportional to the set size). Once the set size equals or exceeds capacity, only one sample is available for each item in memory, so the standard deviation is fixed.

Fitting was achieved by minimizing squared error using a nonlinear optimization algorithm (*fminsearch* in MATLAB) with a range of initial parameter values. We also considered an alternative, bilinear fit to *SD* of the form:

(4) $$\mathrm{SD}(N)=a+b\;\min\{N,K_{\mathrm{SD}}\},$$

as well as a bilinear fit to the estimated number of items in memory:

(5) $$N\times P_{m}(N)=a+b\;\min\{N,K_{P_{m}}\}.$$

Following previous work on discrete capacity models (e.g. Pratte et al., 2017, Rouder et al., 2008, Zhang and Luck, 2008) we allowed our capacity estimates to take on non-integer values.

To test whether the capacity estimates obtained by the two different methods corresponded, we compared the fit of two models, an equality model where $K_{\mathrm{Pm}}=K_{\mathrm{SD}}$ and a linear regression model $K_{\mathrm{Pm}}=\beta_{0}+\beta_{1}K_{\mathrm{SD}}$. Significance values were obtained from an F test based on the residual sums of squares under the two models (equivalent to a likelihood ratio test). Additionally, the Bayesian Information Criterion (BIC) was calculated for each model, and BIC differences are reported in Results. Correlations were calculated using the Pearson correlation coefficient.

### 2.3 Correction for attenuation

Correlations can be corrected for the weakening effect of measurement error using a formula due to Spearman (1904):

(6) $$r_{\mathrm{xy}}^{'}=\frac{r_{\mathrm{xy}}}{\sqrt{r_{\mathrm{xx}}r_{\mathrm{yy}}}},$$

where $r_{\mathrm{xy}}$ is the uncorrected correlation between variables, and $r_{\mathrm{xx}}$ and $r_{\mathrm{yy}}$ are the reliabilities of measurements *x* and *y*. We assessed reliabilities of the two capacity estimates using a bootstrap method (Davison & Hinkley, 1997). For each subject and experiment we generated pairs of $K_{p_{m}}$ estimates and pairs of $K_{\mathrm{SD}}$ estimates, based on resampling the trial-by-trial data with replacement. We repeated this procedure for the whole data set 100 times, and took the mean correlation between pairs as our estimate of reliability for each measurement. Correlations calculated using Eq. 6 are necessarily larger than correlations calculated from the raw data. The principle is that, if *x* and *y* are imperfect measurements of underlying variables x' and y', then $r_{\mathrm{xy}}^{\prime }$ estimates the true correlation between x' and y'.

### 2.4 Bayesian hierarchical model

We additionally fit a Bayesian hierarchical model to data from all participants and experiments simultaneously. In this model, individual participants’ capacity estimates $K_{P_{m}}$ and $K_{\mathrm{SD}}$ were jointly distributed as a bivariate normal:

(7) $$K_{P_{m}},K_{\mathrm{SD}}∼N_{2}(\mu ,\Sigma ),$$

with mean and covariance matrices:

(8) $$\mu =\left(\begin{array}{c} {\overset{‾}{K}}_{P_{m}}\\ {\overset{‾}{K}}_{\mathrm{SD}} \end{array}\right)\;\;\;\Sigma =\left(\begin{array}{cc} \sigma_{K_{P_{m}}}^{2} & \rho \sigma_{K_{P_{m}}}\sigma_{K_{\mathrm{SD}}}\\ \rho \sigma_{K_{P_{m}}}\sigma_{K_{\mathrm{SD}}} & \sigma_{K_{\mathrm{SD}}}^{2} \end{array}\right).$$

The estimated maximum number of items stored was normally distributed with mean $K_{P_{m}}$:

(9) $$\underset{N}{\max}\{N\times P_{m}(N)\}∼N(K_{P_{m}},\sigma_{P_{m}}),$$

and the *SD* at each set size was normally distributed as:

(10) $$\mathrm{SD}(N)∼N\left(\sigma_{1}\sqrt{\min\{N,K_{\mathrm{SD}}\}},\sigma_{\mathrm{SD}}\right)$$

Using JAGS (2003), we fit this model to obtain posterior estimates of population parameters ${\overset{‾}{K}}_{P_{m}},{\overset{‾}{K}}_{\mathrm{SD}},\sigma_{K_{P_{m}}},\sigma_{K_{\mathrm{SD}}}$ and ρ. Code can be found in the Appendix, including specification of priors.

## 3 Results

We examined data from previously published studies testing visual working memory recall at different set sizes. For each study we calculated two estimates of capacity: one based on the frequency of guessing, $K_{P_{m}}$, and one based on the plateau in variability of recalled items, $K_{\mathrm{SD}}$.

**Fig. 2:**
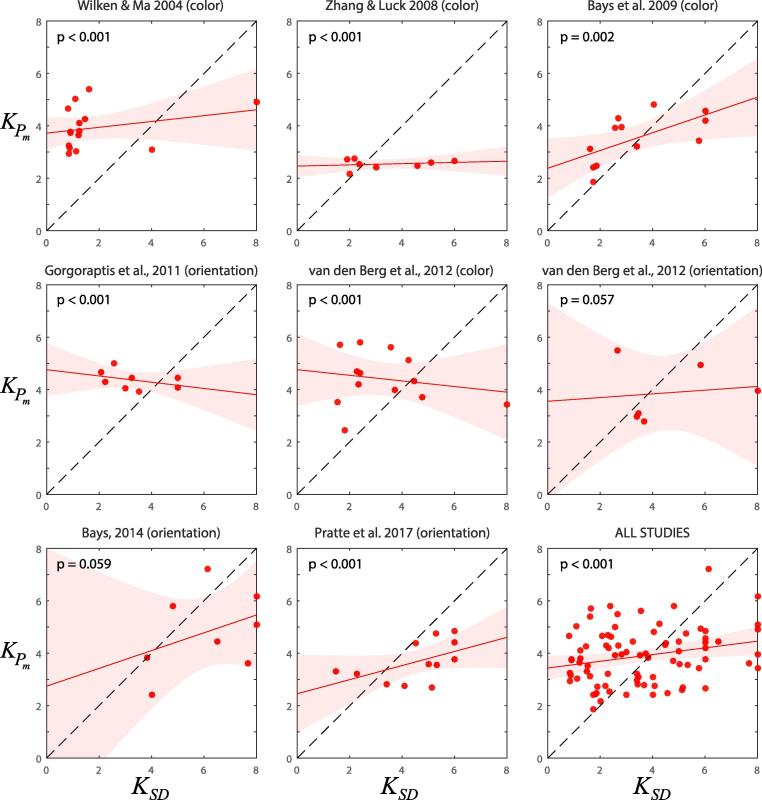
Capacity estimate obtained from the frequency of guessing ($K_{P_{m}}$) as a function of capacity estimated from the plateau in variability ($K_{\mathrm{SD}}$). Each datapoint represents one participant. Each panel presents data from a different study, with data pooled across studies shown bottom right. Red line and colored patch indicate regression line of best fit ± 1 SE. If the capacity estimates correspond they should cluster along the dashed line of equality. P-values indicate significance of a test for deviation from equality. (For interpretation of the references to color in this figure legend, the reader is referred to the web version of this article.)

Scatterplots in Fig. 2 display the relationship between the two estimates, for each individual study and for data pooled across studies (bottom right). If the two estimates matched they would cluster around the dashed equality line in each plot. We found significant evidence against such a correspondence in six out of eight studies (equality model fit significantly more poorly than linear regression model, all p < 0.01; see Methods); the remaining two studies both had p < 0.06 ( mean ΔBIC = 17.9 against equality model across all studies). We observed a significant correlation between the capacity estimates in only one of the eight studies (p = 0.031); the mean correlation across studies was 0.19, explaining 4% of the variance in the data.

Combining data across studies we found strong evidence against a correspondence (p < 0.001; ΔBIC = 107), and a very weak correlation between estimates (p = 0.025, r = 0.25, explaining 6% of the variance). Pooling data from different subject groups in this way may in some circumstances hide a true correlation (Simpson’s paradox; Yarnold, 1996); this can be avoided by standardizing (z-scoring) data from each group: doing so resulted in a slightly reduced, and non-significant, correlation of 0.21, explaining 5% of the variance.

A few participants had one or both estimates equal to the maximum set size tested, implying that larger estimates might have been obtained for these individuals had larger set sizes been used. Excluding these participants from analysis further reduced the correlation between estimates, to 0.09 (explaining < 1% of the variance), indicating that the small correlations reported above are primarily driven by these unreliable estimates. In order to obtain an upper limit on the correlation between estimates we continued to include all participants in subsequent analysis.

The lack of correspondence between estimates might result from poor fits of the plateau model to data. To test this we examined how the absolute difference between estimated capacities varied as a function of the quality of fit of the model (evaluated by the root mean square error). We found no correlation between these values (p = 0.50, r = 0.076, explaining < 1% of the variance), indicating that the correspondence between the two estimates did not improve with the quality of fit. To maximize the data available for fitting, we also calculated capacity estimates for each experiment based on the values of *SD* and $P_{m}$ averaged across subjects: we again found significant evidence against a correspondence between these estimates, now at the experiment level (p = 0.006; ΔBIC = 9.4), and no significant correlation (p = 0.24, r = 0.47, explaining 22% of the variance).

Finally, we found evidence against a correspondence between mean capacity estimates obtained for each experiment (p = 0.022; ΔBIC = 6.1) and no significant correlation (p = 0.39, r = 0.35, explaining 12% of the variance).

### 3.1 Alternative fits

An alternative method for estimating the point of plateau is to use a bilinear fit to *SD* in place of the predictions of the Zhang & Luck model. We applied this model to the present data: we obtained significant evidence against a correspondence in six out of eight studies (p < 0.05) and p = 0.06 for the remaining two (mean ΔBIC = 17.6 against equality model across all studies). We observed significant correlation between estimates in only one out of eight experiments.

It is possible that by taking the maximum estimated number of items stored as a measure of capacity we may have overestimated the true capacity, as the maximization could amplify the noise in each estimate. We therefore also tried fitting estimates of the number of items stored at each set size with a bilinear function and taking the point of plateau as our estimate $K_{P_{m}}$. The results showed significant evidence against a correspondence in six out of eight studies (p < 0.05; mean ΔBIC = 15.2 against equality model across all studies), and significant correlation in only one out of eight (mean r = 0.37, explaining 14% of the variance).

### 3.2 Monte Carlo simulations

To see what results we would expect to observe if the discrete resource model were correct, we simulated behavioral data from the model based on a typical experiment in our data set (10 subjects; 250 trials at each set size) and the means, standard deviations and correlations between parameters obtained from the data fits (K= 3.74 ± 1.3; $\sigma_{1}=$ 17.4° ± 5.3°, correlation coefficient –0.38), and calculated $K_{\mathrm{SD}}$ and $K_{P_{m}}$ from the simulated data in the same way as we had for empirical data. We repeated this procedure 10,000 times. The mean correlation was 0.79 (explaining 63% of the data) and the mean p-value for the test of equality was 0.18. The frequency of obtaining a correlation as low as that obtained from the data (mean r = 0.19) was 0.025, and of obtaining a p-value as low as that obtained from the data (mean p = 0.0148) was 0.205; the probability of obtaining both was 0.009.

A recent study (Pratte et al., 2017) proposed a variant of the discrete resource model, described as a “hybrid” model, in which the precision of representation within each slot varies randomly from trial to trial according to a Gamma distribution (“double stochasticity”; Fougnie et al., 2012, van den Berg et al., 2012). We simulated data based on this model using the Gamma shape parameter obtained in the previous study (τ= 4.4). The mean correlation between capacity estimates was 0.77 (explaining 60% of the variance) and the mean p-value for the test of equality was 0.098. The frequency of obtaining a correlation as low as that obtained from the data was 0.038, and of obtaining a p-value as low as that obtained from the data was 0.357; the probability of obtaining both was 0.014.

### 3.3 Correction for attenuation

Correlations between measurements can be reduced (“attenuated”) by measurement error. We used a resampling (bootstrap) approach to assess individual reliability of the two capacity estimates: we obtained reliabilities of 0.63 for $K_{P_{m}}$ and 0.45 for $K_{\mathrm{SD}}$. From these values and our experiment-average estimate of correlation we calculated a corrected-for-attenuation correlation of 0.34, explaining 11% of the variance.Thus, even when the effects of measurement error were taken into account, no meaningful correlation between estimates was obtained.

### 3.4 Bayesian hierarchical modeling

**Fig. 3:**
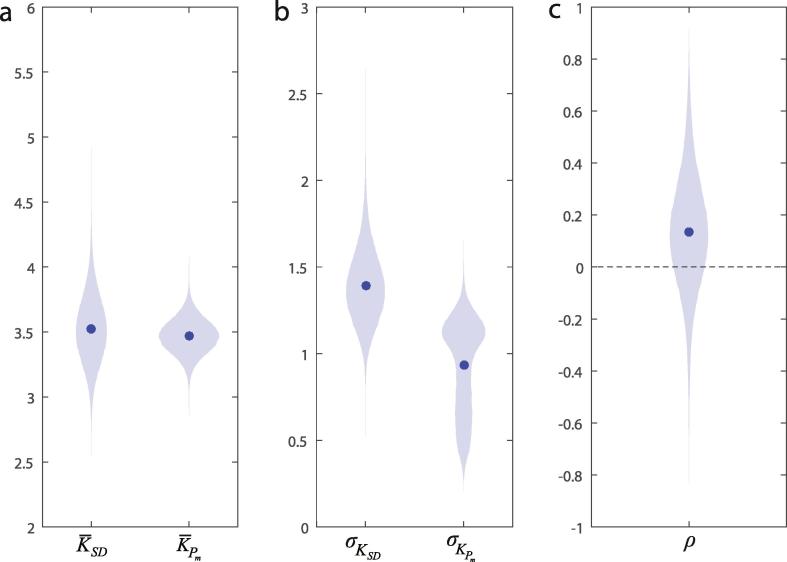
Bayesian hierarchical model parameters. (a) Posterior distributions of the population mean capacity derived from *SD* (left) and $P_{m}$ (right). Data points indicate posterior means. (b) Posterior distributions of the population standard deviation of capacity derived from each source. (c) Posterior distribution of the correlation between capacity estimates derived from each source.

To further improve the quality of fits we applied a Bayesian hierarchical approach, in which each participant’s capacity estimates $K_{\mathrm{SD}}$ and $K_{P_{m}}$ were assumed to be drawn from a bivariate distribution with unknown mean, variability, and correlation between the estimates. This approach uses the distribution of group-level estimates to constrain extreme values in the parameters at the individual level, resulting in more reliable estimates (Rouder & Lu, 2005). The resulting parameter estimates are shown in Fig. 3.

Confirming results of individual fits, the estimated (mean posterior) correlation was 0.14, explaining 2% of the variance (Fig. 3c). The posterior 95% highest density interval [–0.33, 0.61] encompassed zero, indicating no significant evidence of a correlation between the two capacity estimates.

## 4 Discussion

We have shown that two different calculations that should both estimate an individual’s capacity under the discrete resource model produce incompatible results. This represents strong evidence against the concept of discrete representations in visual working memory.

Simulations confirmed that our tests were sensitive to the correspondence between estimates and that the obtained results were highly unlikely under the discrete resource model. Two attempts to bolster the correlation between capacity measures by reducing the effect of measurement noise, either applying a correction to the correlation calculation or fitting a Bayesian hierarchical model, both failed to produce a meaningful correlation between estimates.

We also examined a recently proposed “hybrid” model (Pratte et al., 2017) that combines variability in precision with a discrete resource framework (with the result that it is no longer discrete). This modification had minimal effect on the predicted correlation between capacity estimates, and the observed correlation remained highly unlikely under this model (in contrast, the equality test proved less robust to this modification).

In general, any model that seeks to explain the decline in precision with set size in terms of a set of discrete representations (e.g. slots) will predict a strong relationship between capacity measures obtained from variability and guess frequency, because both are ultimately determined by the number of representations available. The absence of such a relationship provides compelling evidence against discrete representations. In contrast, the present results are potentially consistent with an account in which a maximum capacity exists independently of the decline in precision, e.g. a continuous resource model in which there is an upper bound on the number of items the resource can be allocated to. However, proponents of such a model would need to explain why statistics of the error distribution (e.g. standard deviation or kurtosis of errors) all change smoothly and continuously with set size (see e.g. Bays, 2015), with no indication of a change in response behavior at the capacity limit.

A recent study by Adam, Vogel, and Awh (2017) examined performance on a whole-report task, in which participants reproduced all items in a memory display in turn, in an order of their choosing. The authors claimed to find evidence for an upper bound on the number of items stored. A full assessment of their results is beyond the scope of the present paper, but we would point out that their claims are based on data showing that, as participants report the items on a trial in sequence, their responses become gradually more variable until they are indistinguishable from noise. Rather than demonstrating any change in performance that would indicate a participant had reached their maximum capacity, the authors used statistical methods that arbitrarily defined a level of variability beyond which responses were considered uniform (and hence pure guesses). The authors did not compare fits of working memory models to their data, but we see nothing that would obviously present a challenge to resource models with no fixed capacity.

Our approach in this study has been to assume the discrete resource model is true and demonstrate that this leads to an internal inconsistency. In doing so we have intentionally ignored a number of other criticisms that have been leveled at the model, including the fact that many of the errors ascribed to guessing are in fact “swap” errors in which a non-target item is reported (Bays et al., 2009, Emrich and Ferber, 2012, Rerko et al., 2014, Schneegans and Bays, 2017); and the observation that plausible models of internal coding do not predict a von Mises distribution of error for items in memory, as assumed by the discrete resource model (Bays, 2014).

We also allowed the capacity estimates to take on non-integer values, in order to give the discrete resource model the best chance of fitting the data. While seemingly contradictory to the discrete item concept, such values could be interpreted under a model in which an individual’s capacity varies randomly from trial to trial (Taylor, Thomson, Sutton, & Donkin, 2017): the non-integer value would indicate the mean capacity. As most, if not all, previous modeling work by proponents of the discrete resource hypothesis has allowed non-integer capacities (e.g. Pratte et al., 2017, Rouder et al., 2008, Zhang and Luck, 2008), this moment-to-moment variability in capacity appears to be an unspoken assumption of the discrete model. We hope that any future iterations of the discrete resource model will make this assumption explicit.

A previous study (van den Berg & Ma, 2014) argued that comparing models using summary statistics, including capacity estimates of the kind examined here, is an inferior method of assessing models compared to likelihood-based comparisons on individual trial data. Although their analysis was based on data from another research group that, unfortunately, we now know to have been falsified, we nonetheless largely agree with their conclusions. Indeed when formal model comparisons have been conducted on recall data they have almost without exception demonstrated a substantial advantage for continuous over discrete resource models (e.g. Bays and Taylor, 2018, Bays, 2014, Keshvari et al., 2013, van den Berg et al., 2014, van den Berg et al., 2012). However, we would argue that converging evidence from different methods provides the strongest argument against a theory, and in the present study we have taken an alternative approach by assaying a simple intuitive test of the discrete hypothesis.

It is important to note that a correlation between capacity estimates would not have represented strong evidence for the discrete resource model: the two estimates are derived from the same data, so for them to exhibit a correlation would not be particularly surprising; instead, the discrete resource model makes the clear prediction that they should be equal. Nonetheless, we observed only a very weak correlation, explaining less than 10% of the variance in the estimates. Our analysis found strong and consistent evidence across studies that the discrete resource model does not provide a self-consistent estimate of capacity.

## Data availability

All data associated with this article can be found at https://osf.io/xvz2y/.

## Figures and Tables

**Table:** 

data {
for (i in 1:nsubj) {
for (j in 1:nn) {
KK[i,j] = ns[1,j]∗pm[i,j]
}
K_pm[i] <- max(KK[i,])
}
}

model {
for (i in 1:nsubj) {
for (j in m[i,1:nm[i,1]]) {
sd[i,j] ∼ dnorm(mu[i,j], tau) T(0,)
mu[i,j] <- sd1[i] ∗ sqrt(min(ns[i,j], max(K_est[i,1], 0)) )
}
sd1[i] ∼ dnorm(sd1_mu, sd1_tau) T(0,)
K_pm[i] ∼ dnorm(K_est[i,2], tau_pm) T(0,)
K_est[i,1:2] ∼ dmnorm(K_mu[1:2], K_omega[1:2, 1:2])
}
tau ∼ dgamma(10^∧^-3, 10^∧^-3)
tau_pm ∼ dgamma(10^∧^-3, 10^∧^-3)

sd1_mu ∼ dnorm(10.0, 10^∧^-3) T(0,)
sd1_tau ∼ dgamma(10^∧^-3, 10^∧^-3)

R[1,1:2] <- c(1, 0)
R[2,1:2] <- c(0, 1)
K_omega ∼ dwish(R, 2)

for (i in 1:2) {
K_mu[i] ∼ dnorm(4, 10^∧^-3) T(0,)
}
}

**Table:** 

pm[i,j]	$P_{m}$ estimate for subject *i* and set size *j*
sd[i,j]	*SD* estimate for subject *i* and set size *j*
nsubj	Number of participants
nn	Maximum set size
nm[i,1]	Number of set sizes tested for participant *i*
m[i,j]	*j*th set size tested for participant *i*
ns[i,j]	index array of set sizes in form e.g. [1,2,nan,4,nan,nan,nan,8]

## Appendix A

The following is JAGS code describing the Bayesian hierarchical model.

Definitions:

## Footnotes

1 Note that the correlation between these variables was previously examined in several papers by Anderson, Vogel & Awh that have subsequently been retracted.
